# BAD, a Proapoptotic Protein, Escapes ERK/RSK Phosphorylation in Deguelin and siRNA-Treated HeLa Cells

**DOI:** 10.1371/journal.pone.0145780

**Published:** 2016-01-08

**Authors:** Samra Hafeez, Mahwish Urooj, Shamiala Saleem, Zeeshan Gillani, Sumaira Shaheen, Mahmood Husain Qazi, Muhammad Imran Naseer, Zafar Iqbal, Shakeel Ahmed Ansari, Absarul Haque, Muhammad Asif, Manzoor Ahmad Mir, Ashraf Ali, Peter Natesan Pushparaj, Mohammad Sarwar Jamal, Mahmood Rasool

**Affiliations:** 1 Institute of Molecular Biology and Biotechnology and Center for Research in Molecular Medicine, the University of Lahore, Lahore, Pakistan; 2 Center of Excellence in Genomic Medicine Research, King Abdulaziz University, Jeddah, Saudi Arabia; 3 College of Applied Medical Sciences, King Saud Bin Abdulaziz University for Health Sciences, National Guards Health Affairs, Riyadh, Saudi Arabia; 4 King Fahd Medical Research Center, King Abdulaziz University, Jeddah, Saudi Arabia; 5 Department of Biotechnology, BUITEMS, Quetta, Pakistan; 6 College of Applied Medical Science, Al Majmaah University, Majmaah City, Saudi Arabia; Rosalind Franklin University of Medicine and Science, UNITED STATES

## Abstract

This study has been undertaken to explore the therapeutic effects of deguelin and specific siRNAs in HeLa cells. The data provided clearly show the silencing of ERK 1/2 with siRNAs and inhibition of ERK1/2 with deguelin treatment in HeLa cells. Additionally, we are providing information that deguelin binds directly to anti-apoptotic Bcl-2, Bcl-xl and Mcl-1 in the hydrophobic grooves, thereby releasing BAD and BAX from dimerization with these proteins. This results in increased apoptotic activity through the intrinsic pathway involved in rupture of mitochondrial membrane and release of cytochrome C. Evidence for inhibition of ERK1/2 by deguelin and escape of BAD phosphorylation at serine 112 through ERK/RSK pathway has been further fortified by obtaining similar results by silencing ERK 1/2 each with specific siRNAs. Increase in BAD after treatment with deguelin or siRNAs has been interpreted to mean that deguelin acts through several alternative pathways and therefore can be used as effective therapeutic agent.

## Introduction

A number of natural compounds have been shown to have apoptotic activity against a variety of cancers [1]. The chemotherapeutic activity of deguelin, a retinoid, extracted from *Mundulea Sericea* (Leguminosae) is presently under intensive investigation [2–4]. Strong antitumor activity of deguelin has been demonstrated both in vitro and in vivo [2,5]. Substantial evidence is now available to show that deguelin inhibits PI3K-Akt pathway. The inhibitory effect of deguelin on RAS-MAPK pathway has also been demonstrated In one report data have been provided in which deguelin has been shown to suppress I*κ*k, I*κ*b and NFκB, thereby reducing the synthesis of Bcl-2 family of anti-apoptotic proteins in Human Bronchial Epithelial (HBE) cells [1]. In Mouse Myeloma cells it inhibits growth by inducing apoptosis [6]. In gastric cells deguelin induces apoptosis through caspase-9 and caspase-3 pathway [7,8]. In head and neck squamous cell carcinoma, deguelin inhibits the phosphorylation of Akt which leads to apoptosis and autophagy [2]. More recent evidence indicates that BAD is phosphorylated at Ser-112 by the RAS-MAPK pathway, affected through ERK 1/2 and mediated by p90 Ribosomal S6 kinase (RSK) [7,9]. Furthermore, JNK-1, RSK-2 and MSK-1 have also been implicated in the phosphorylation of BAD at Ser-112 [9,10]. Additionally, deguelin is claimed to induce apoptosis by down regulating inhibitors of apoptosis proteins (IAPs) and survivin [11].

A critical balance is maintained between cell survival and apoptosis by Bcl-2 family proteins. The anti-apoptotic Bcl-2, Bcl-xl and Mcl-1 antagonize proapoptotic proteins and prevent their function of initiating apoptosis by oligomerisation on the outer mitochondrial membrane (OMM), which leads to pore formation and release of cytochrome C, with subsequent activation of caspase 9 and caspase 3 [12–14]. Whether deguelin induces apoptosis through its binding with hydrophobic groove of anti-apoptotic proteins, especially, Bcl-2 and Bcl-xl is not known. Nor is it clear whether deguelin directly binds to ERK 1/2 and down regulates its function of phosphorylating RSK. The inhibition of ERK 1/2 therefore prevents phosphorylation of BAD at Ser-112 thereby enabling BAD to escape ubiquitination. More recently, because of collateral damage caused by various chemotherapeutic drugs, a number of new mechanisms are being used for silencing various substrates of RAS-MAPK pathway. This silencing of a specific substrate for example MAP kinase can be affected using ERK1 and ERK2 specific siRNAs.

In this report we are providing evidence that (a) deguelin induces apoptosis by binding to anti-apoptotic proteins (Bcl-2, Bcl-xl and Mcl-1) in the hydrophobic groove as demonstrated by bioinformatics tools and (b) suppression of ERK 1/2 expression by deguelin which is supported by specific silencing of ERK 1 and ERK 2 using siRNAs. The effect of silencing ERK 1 and ERK2 by siRNA and effect of deguelin treatment on ERK 1/2 expression has been documented through blotting of relevant proteins.

## Materials and Methods

### Materials

DMEM (Dulbecco modified eagle medium), L-glutamine (200mM), Antibiotics (Penstrep), Fetal Bovine Serum (FBS), Versene-EDTA and Phosphate Buffered Saline (PBS) were obtained from GIBCO-Invitrogen, USA. Deguelin was purchased from Tocris Life Sciences, UK, with a purity of > 97%. siRNAs specific for ERK-1 or ERK-2 were obtained from Santa Cruz, USA. Effectene transfection reagent was purchased from Qiagen, USA. All primary antibodies were purchased from Biovision, USA. TMB (Tetramethylbenzidine) substrate was purchased from Sigma, USA. Qubit Protein Quantification kit and Pre-stained protein markers were obtained from Invitrogen, USA. Tissue culture flasks and 6-well culture plates were purchased from Oxygen life sciences, California, USA.

### Methods

#### Cell culture

HeLa cell line was kindly provided by the School of Biological Sciences (SBS), University of Punjab, Lahore and was cultured under standard conditions in DMEM with 10% FBS in six well plates. Cells were observed daily for growth. When plates were 80–90% confluent, cells were transfected either with ERK-1 or ERK-2 siRNA. In each case one plate served as a control.

#### Transfection

15μl (1 μg, 1.5 μg or 2 μg) of both ERK1 and ERK2 siRNAs were mixed with 225μl EC buffer and 12μl of enhancer in separate microfuge tubes. After gentle mixing and 5 min incubation, 15μl of effectene transfection reagent was added to both microfuge tubes and incubated for 15 min. Old medium of the plates was replaced with 2.7 ml of fresh DMEM. 300μl DMEM was added into both microfuge tubes and total contents of tubes were poured into respective plates. Both plates were then incubated at 37°C for 48 hrs. After 48 hours, lysates of both were prepared using RIPA lysis protocol.

#### MTT assay

The assay was carried out using standard protocol to check percentage of cell viability after Deguelin treatment on HeLa cells. Cells were plated at a density of 5x10^3^ in 96-wells plate and allowed them to settle overnight. Various concentrations of Deguelin, 1μM, 5μM, 10μM, 20μM and 40μM, were administered for a time period of 24 hours.

#### Deguelin treatment

Cells for deguelin treatment were prepared under aseptic conditions. Cells were plated at a density of 1x 10^6^ cells per plate (100mm). One plate served as a control and the remaining plates were subjected to various doses (1μM, 5μM, 10μM, 20μM and 40μM) of deguelin dissolved in 10mM of DMSO for 24 hours.

#### Radio immuno precipitation assay (RIPA) lysis

After the termination of experiments plates were placed on ice in the hood. Cells were scrapped from each plate and shifted to 15ml falcon tubes. Falcon tubes were centrifuged at 6000 rpm for 5 min at 4°C to obtain pellet. The pellet was washed twice with PBS with repeated centrifugations at 6000 rpm. RIPA lysis cocktail (1.5μl phenyl methyl sulphonyl fluoride PMSF, 1.5μl sodium orthovendate, 1.5μl protease inhibitor and 150μl RIPA lysis buffer containing 1xTBS, 1% Nonidet P-40, 0.5% sodium deoxycholate, 0.1% SDS and 0.004% sodium azide) was then added to each falcon tube and incubated on ice for 30 min. Material was then centrifuged at top speed (14,000 rpm) for 10 min at 4°C to separate whole cell lysate from debris. Supernatant was used for quantification of proteins and further analysis by polyacrylamide gel electrophoresis (PAGE). Equal quantities of proteins were used for protein blotting.

#### Protein blotting

Aliquots of lysates were denatured after adding 2XSDS loading Buffer and resolved by 12% SDS polyacrylamide gel electrophoresis (PAGE). Nitrocellulose membrane (BioRad, USA) was used for blotting the proteins from the gel onto the membrane using TetraProtean^®^ system at 200 volts. The membrane was blocked with 5% Bovine Serum Albumin (BSA) in phosphate buffer saline with 0.1% Tween 20 (PBST) at room temperature for 2 hrs. The membrane was then exposed to the specific primary antibody (1:500) diluted in BSA and incubated overnight in cold room on shaker. The next day membrane was washed with PBST, 5 times for 5 min and then incubated with suitable secondary HRP conjugated antibody. Membrane was then washed again with PBST, 5 times for 5 min and visualized by using TMB as substrate in gel documentation system (BioRad CA, USA) [15].

#### Binding of Deguelin

Using various informative tools the binding of deguelin was studied with the possible site of binding with Bcl-2, Bcl-xl and Mcl-1 (Anti-apoptotic proteins). Crystallized structures of the respective target proteins were downloaded from RCSB protein data bank in the form of PDB file. The respective proteins having NMR structures are 1GJH, 1BXL, 2KBW.

#### Preparation of ligands

PubChem database was used to acquire the sdf file of 1 mimic. Open-babel was used to convert the sdf file to pdb file to obtain its 2D structure and converted into 3D structures. The 3D structures were minimized using hyperchem’s MM+force field. Pdb file of mimic was imported into MGL tools to check torsion angles and convert it into PDBQT format to store its atomic coordinates, partial charges and a description of the rigid and rotatable parts of the molecule.

#### Pre-processing of the target protein

Individual protein was loaded into MGL tools to refine the structure. First it was checked to remove any heteroatoms in the PDB file in order to correct the chemistry of the target protein. All the non-polar hydrogens were merged and the water molecules were removed from the receptor file and their partial charges were added to the corresponding carbon atoms. The receptor PDB file was transformed into the PDBQ format file containing the receptor atom coordinates, partial charges and salvation parameters.

#### Docking

Docking simulations were performed selecting VINA and AutoDock docking server. Grids calculations were set up and maps were calculated with a program AUTOGRID. The grid maps were centered on the ligand binding site with a dimension of 90 × 90 × 90 points. A grid spacing of 0.325 Å and the default AutoDock parameter settings were used for docking.

MGL is program which is used to visualize the protein, generate grids for binding site and analyze the results. PDB file of proteins and ligands were converted to PDBQT by applying Gasteiger charges. An exhausted search was performed using Lamarckian Genetic Algorithm (LGA) which enables docking to be very accurate.

## Results

### MTT assay

**Fig 1:**
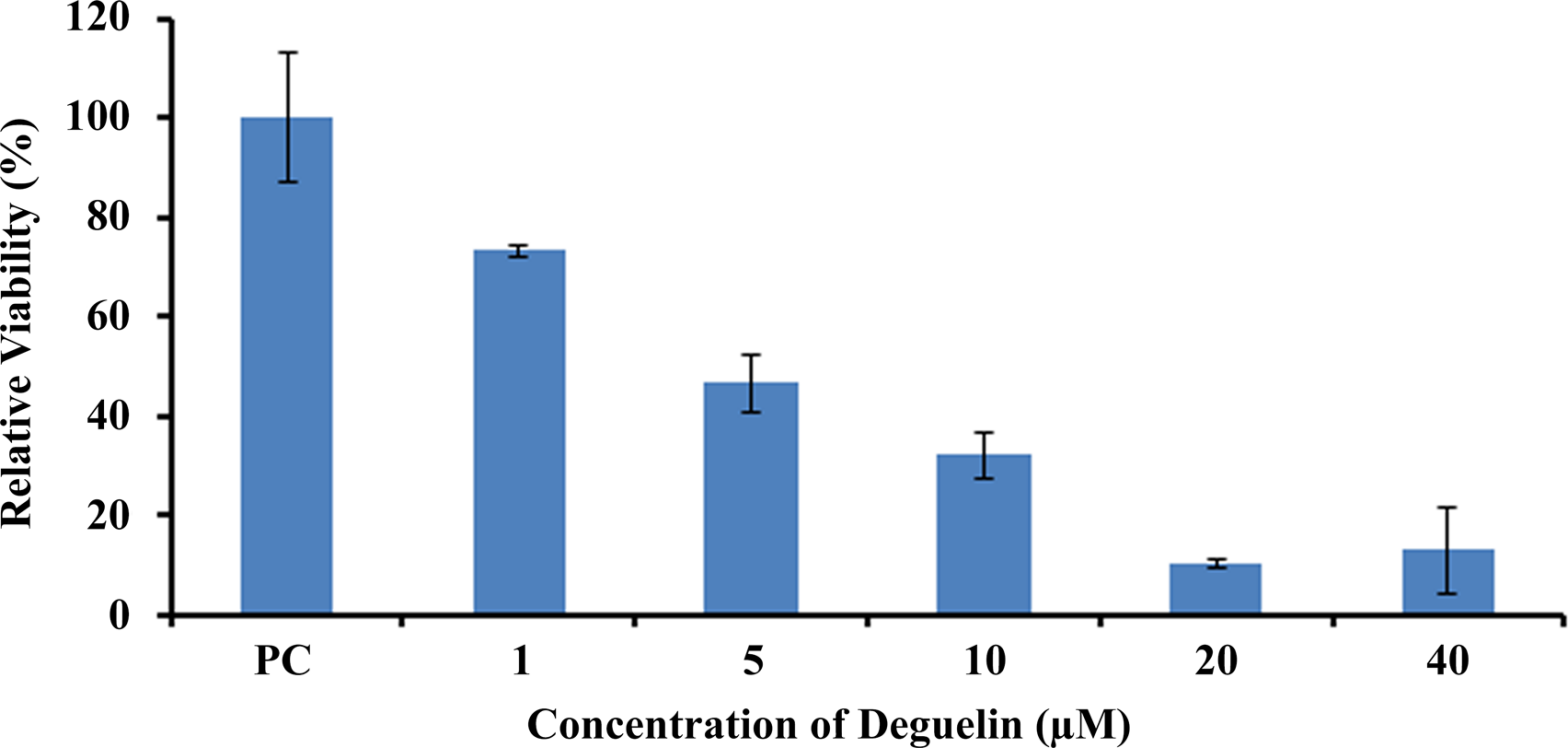
MTT Assay in HeLa cells. The MTT assay was carried out after plating HeLa cells at a density of 5000 cells/well in 96 well plates. The cells were treated with different doses of deguelin. It shows a regression curve of cell viability at the indicated doses.

In order to access the therapeutic effect of deguelin in vitro, 5x10^3^ HeLa cells per well were plated in 96 well plates. Deguelin was added to the culture cells in appropriate dilution ranging from 1μM to 40μM. The results obtained show the average of three replicates, it may be observed that deguelin has remarkable in vitro therapeutic efficacy (Fig 1).

### Docking of deguelin

**Fig 2:**
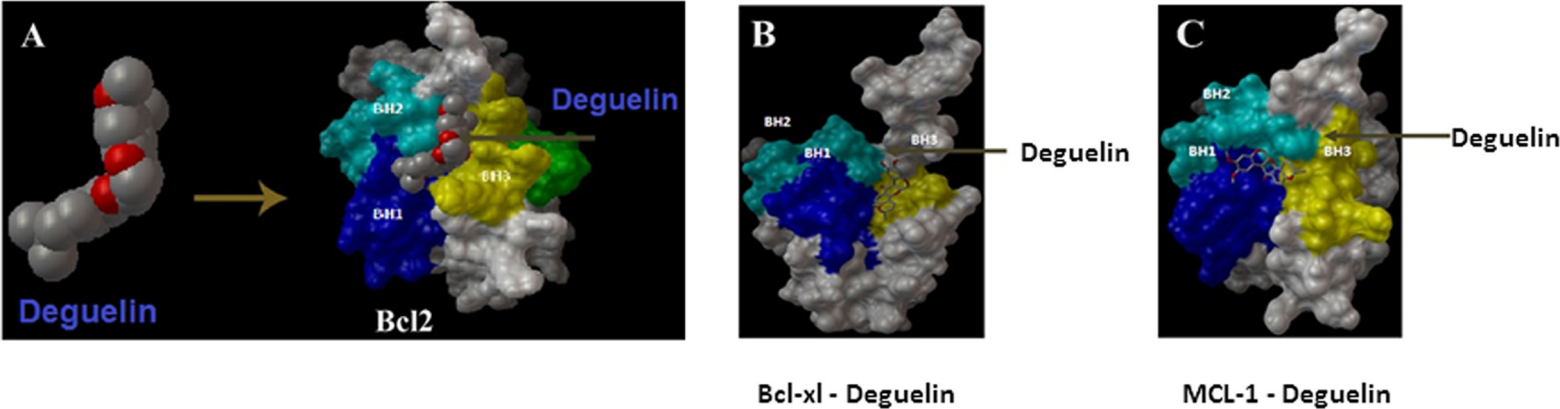
Docking of deguelin on anti-apoptotic proteins. (A) Structure of deguelin arrow indicates the binding of deguelin on Bcl-2 (B) The docking of deguelin on BH3 domain of hydrophobic group of anti-apoptotic protein Bcl-xl (C) Binding of deguelin on MCL-1 (D) Binding of deguelin molecule on ERK-1 near the ATP binding site. Docking was carried out using bio-informative tools as described in materials and methods.

In order to find out whether deguelin can bind anti-apoptotic proteins (Bcl2, Bcl-xl, Mcl-1) in the hydrophobic groove, and can thus inhibit their ability to oligomerize with pro-apoptotic proteins. Hydrophobic groove, comprised of BH1, BH2 and BH3 domains, engages the pro-apototic proteins by providing docking sites. Deguelin can have the ability to cease their hydrophobic groove and release pro-apoptotic proteins. As a consequence they can work independently to induce apoptosis by releasing cytochrome c. Deguelin was docked on to the respective anti-apoptotic proteins using the bioinformatics tools described under materials and methods. The data obtained are shown in Fig 2A, 2B and 2C.

It may be seen that deguelin binds to Bcl2 and Mcl-1occupying the hydrophobic groove and binding with BH1, BH2 and BH3 domains. However, in Bcl-xl it binds only with BH3 domain. The binding of specific amino acids of the ligand (Deguelin) with BH1, BH2 and BH3 domains of receptor proteins are shown in Table 1.

**Table 1:** The binding of Deguelin (ligand) with specific amino acids present in BH1, BH2 and BH3 domains of receptor proteins.

S.No	Receptor	Domains	Deguelin docking Site	Binding energy (Kcal/mol)	Specific amino acid(s)
**1**	MCL-1	BH1, BH2, BH3	BH1,BH2,BH3	-6.7	ASN260,TRP261,GLY262,VAL265,PHE315,PHE318,PHE319
**2**	BCL2	BH1, BH2, BH3, BH4	BH1,BH2,BH3	-7.0	THE96,ALA100,GLN99,ASP103,PHE104,TRP144,GLY145,VAL148,TYR202,GLX203,PRO204
**3**	BCl-XL	BH1, BH2, BH3, BH4	BH1,BH2,BH3	-8.3	ALA93,GLU96,PHE97,ARG100,TRY101,GLY138,VAL141,ALA142,LEU194,TYR195,ALA199,AUA200,SER203
**4**	BAK	BH1, BH2, BH3	NA	NA	NA
**5**	BAX	BH1, BH2, BH3	NA	NA	NA
**6**	BID	BH3	NA	NA	NA

**Fig 3:**
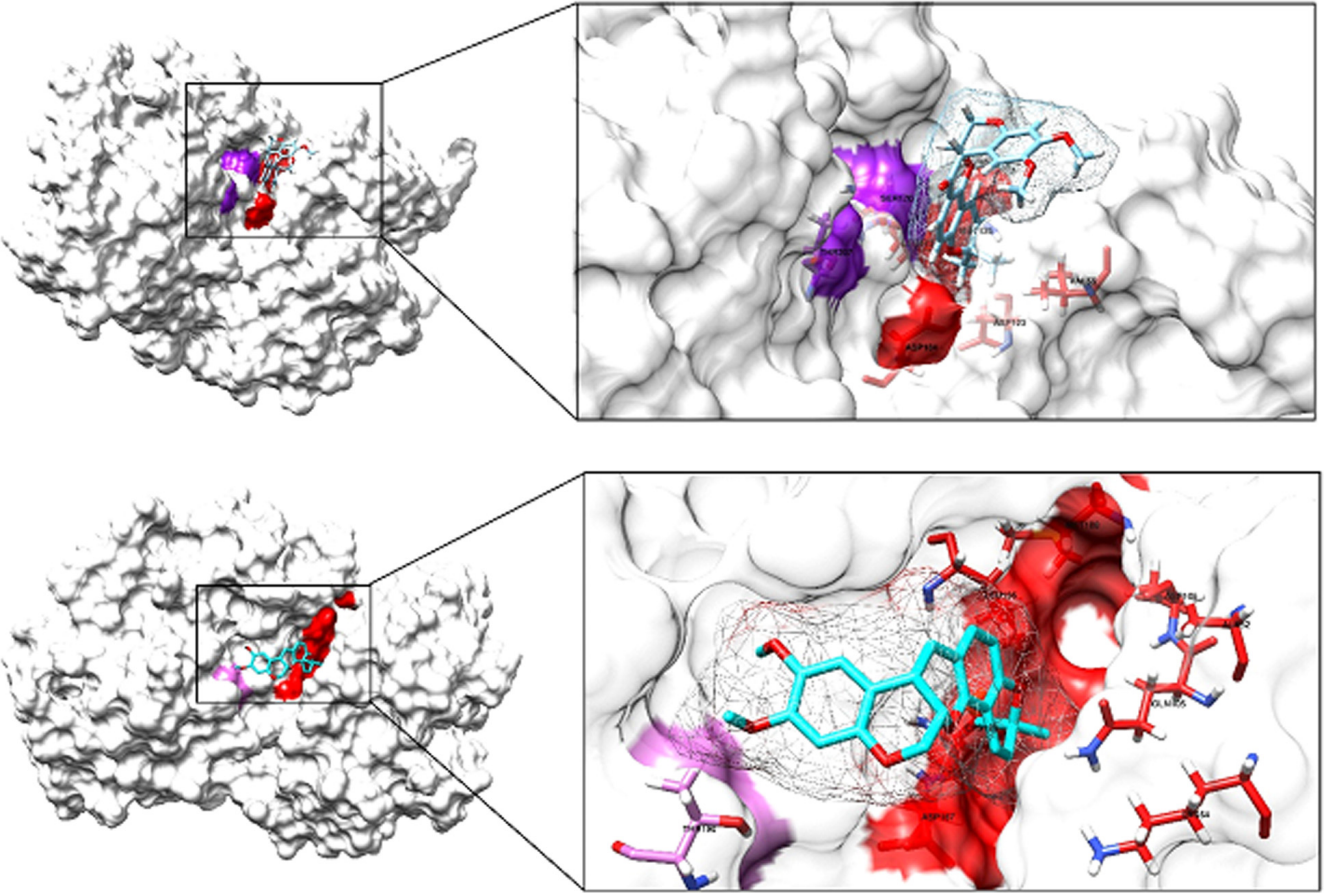
The binding of deguelin molecule on ERK-1 and ERK-2 near the ATP binding site. Docking was carried out using bio-informative tools as described in materials and methods. It may be observed that the molecule binds to ERK1/2 in the ATP pocket, thereby, preventing the phosphorylation of ERK.

Deguelin was also docked on to ERK1 and ERK2 (Fig 3). It may be observed that the molecule binds to ERK1/2 in the ATP pocket, thereby, preventing the phosphorylation of ERK. Absence of Phosphorylation in ERK has implications for the phosphorylation of its substrates, including Ribosomal S6 Kinase (RSK). Simultaneously, when these samples were blotted for Bad there was substantial increase as documented by the intensity of the blot. At the same dose level there was appreciable decrease in Bcl-xl, with little change in Bax. Cytochrome-C which is a positive marker of initiation of apoptosis is markedly increased at the dose level used. When total ERK was blocked by using 1μM and 10μM concentrations of Deguelin, ERK1/2 completely disappeared from blots in the presence of 10μM Deguelin.

### Silencing of ERK1/2 using specific siRNAs

**Fig 4:**
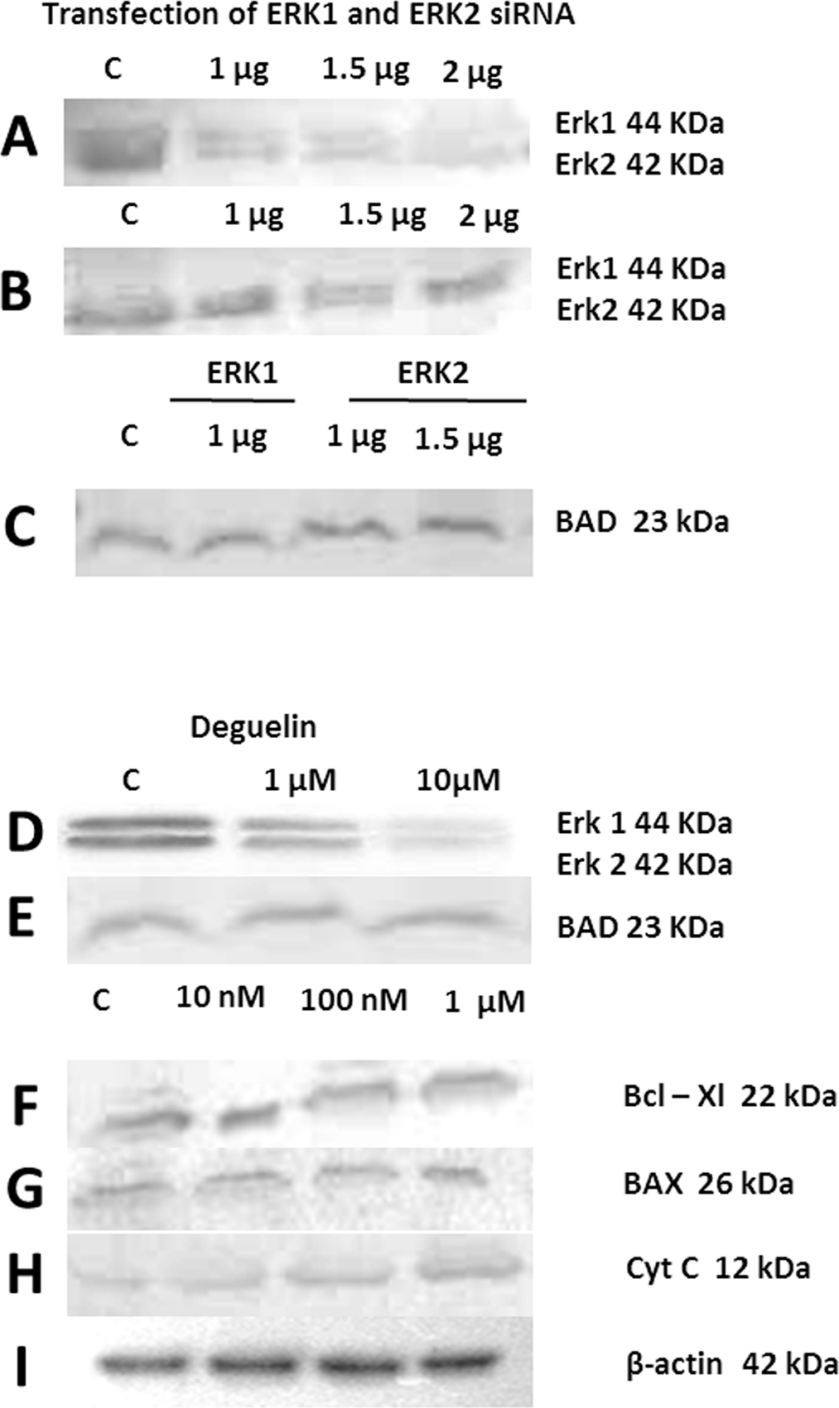
The figure shows the blotting of various proteins isolated from normal cells and those treated with various doses of either deguelin or transfected with siRNAs specific for silencing of ERK1/2. (A) Cells transfected with various doses of ERK-1 specific siRNA. (B) Cells transfected with various doses of ERK-2 specific siRNA. (C) BAD expression in cells transfected with either ERK-1 or ERK-2 siRNAs. (D) ERK-1 and ERK-2, (E) BAD, (F) Bcl-xl, (G) BAX and (H) Cytochrome-C levels in cells treated with various doses of deguelin. (I) The house-keeping control (β-actin).

In another set of experiments ERK1 and ERK2 were silenced using specific siRNA for each. In this experiment HeLa cells were used and transfected with various doses of siRNA for ERK-1 (Fig 4A) and ERK-2 (Fig 4B) and it may be observed that ERK-1 is significantly silenced in Fig 4A and ERK-2 is eliminated significantly in Fig 4B when transfected with a dose of 2μg. In Fig 4C, BAD was probed in ERK-1 and ERK-2 specific siRNA transfected HeLa cells. A minor reduction in total BAD was observed when ERK-1 was silenced while in case of ERK-2 silencing there was no change in total BAD protein. Cell treated with deguelin at dose levels of 1μM and 10μM showed progressive reduction in both ERK-1 and ERK-2 proteins level (Fig 4D). Deguelin treatment at 1μM and 10μM causes possible increase in BAD (Fig 4E). There was no change in Bcl-xl at various dose levels with deguelin treatment (Fig 4F); similarly there was no change in BAX (Fig 4G). While a remarkable increase was seen in Cytochrome-C levels with deguelin treatment (Fig 4H). β-actin was used as housekeeping control for normalization (Fig 4I).

The results of protein blotting obtained are shown in Fig 4 and S1 File (Figs A-F). Like that of deguelin, the silencing of total ERK1 and ERK2 resulted in increased blotting density of proapoptotic BAD. These results confirm that inhibition of ERK either with deguelin or silencing with siRNAs result in increase in BAD.

## Discussion

A number of studies have demonstrated that deguelin, a retinoid, inhibits the Akt pathway in a number of cancers [2,4,6,11,16]. In breast cancer cells however, deguelin inhibits the phosphorylation of p44/42 MAPK (ERK1/2) [11]. Exploring the RaS mitogen activated pathway, it has been shown that BAD is phosphorylated at ser-112 by ERK1/2 through the mediation of p90 RSK. This information established a link between the survival effects of cytokines with the cell death machinery (Roux and Blenis., 2004). Phosphorylation of BAD at serine-136 by Akt prevents it from heterodimerizing with anti-apoptotic proteins. Furthermore, Phosphorylation of BAD by ERK1/2 on Ser-112 leads to ubiquitination of this protein through 14-3-3 protein [7].

In our data we have noted that administration of deguelin resulted in silencing ERK1/2 possibly through competing with its ATP binding site. Using bio-informative tools, this was confirmed from binding of deguelin with ERK1/2 (Fig 3). Accordingly, we have concluded that binding of Deguelin with ERK 1/2 prevents the phosphorylation of BAD resulting in increase of total BAD as is evident from our protein blots (Fig 4). In the absence of any previous data on the use of siRNA in the silencing of ERK, we have fortified the results obtained with Deguelin by silencing ERK 1/2 with siRNAs specific for each isoform. In our studies similar results obtained for BAD (Fig 4) as with Deguelin. This part of the study, in essence, demonstrates that by silencing ERK 1/2 with either of the two molecules, BAD escapes phosphorylation and ubiquitination. This is documented in Fig 4. The increase in cytochrome c observed due to the inhibition of ERK by Deguelin treatment (Fig 4H) that gives indication that Deguelin also causes apoptosis through the activation of caspases 3, the executioner caspases.

Using bio-informatics tools, we have also observed the docking of Deguelin onto anti-apoptotic Bcl-2 family proteins, namely, Bcl-2, Bcl-xL and Mcl-1. This information was obtained in order to find out whether Deguelin also acts through binding with the hydrophobic groove of these proteins. Our data clearly shows that this retinoid binds to BH1, BH2 and BH3 domains of the hydrophobic groove thereby preventing hetero-dimerization with apoptotic proteins, BAX and BAD (Fig 3A, 3B and 3C). This conclusion is supported by increase of cytochrome c (Fig 4H). Taken together, we can conclude that Deguelin is an effective therapeutic agent in causing apoptosis in HeLa cells acting both through RAS-MAPK pathway and also by binding to anti-apoptotic proteins, thereby releasing the apoptotic proteins for oligomerization on the mitochondrial membrane and release of cytochrome c. Furthermore, silencing of ERK 1/2 by Deguelin or siRNA gives identical results with regard to the escape of BAD from phosphorylation and thus ubiquitination.

This observation led us to the postulate that silencing of ERK1/2 may result in concomitant increase in pro-apoptotic BAD, since it will not be phosphorylated at ser-112 and would thus escape ubiquitination. This postulate was further fortified by directly silencing ERK 1 and 2 using specific siRNAs for each. This also resulted in increase of BAD (Fig 4). Interesting enough, with Deguelin, we also noted an increase in cytoplasmic cytochrome C indicating thereby that this molecule causes apoptosis through the intrinsic pathway involving BAX and BAD. Further, our binding studies of deguelin with anti-apoptotic proteins Bcl-2, Bcl-xL and Mcl-1 in the hydrophobic groove indicate that BAX and BAD are spared from dimerization with these anti-apoptotic proteins. In a recent review it has been argued that of the various substrates of RSK, BAD, an antagonist of Bcl-2/Bcl-xl is phosphorylated at ser-112 [17].It is therefore apparent that ERK phosphorylation of BAD is mediated through RSK. In our data we have observed elevation of BAD as shown by protein blotting. This means that BAD escapes phosphorylation by RSK mediated ERK phosphorylation. This has been observed both with deguelin treatment of HeLa cells as well as silencing of ERK by specific siRNAs for ERK-1 and 2. The differential effect of ERK1/2 silencing by siRNA specific for each ERK species indicates that silencing of ERK-2 has a more profound effect on the escape of BAD phosphorylation by RSK mediated phosphorylation of ERK-2. Further, our observation that deguelin promotes apoptosis through its binding with anti-apoptotic proteins Bcl-2, Bcl-xl and Mcl-1 in the hydrophobic groove indicates that it promotes apoptosis through several alternative routes. First, through preventing phosphorylation of Akt, Secondly, by directly down regulating ERK1/2 as is evident from our data and finally by binding to anti-apoptotic proteins thereby releasing pro-apoptotic Bax and BAD from dimerization with these proteins.

## Supporting Information

S1 File Western Blotting Gel Pictures. Bcl-xl (Figure A). Bax (Figure B). BAD (Figure C). Cytochrome C (Figure D). ERK 1/2 (Figure E). β-actin (Figure F).
(PPTX)
