# Supplementary material for: BAD, a Proapoptotic Protein, Escapes ERK/RSK Phosphorylation in Deguelin and siRNA-Treated HeLa Cells
Source: PLoS One. 2016 Jan 8;11(1):e0145780. doi: 10.1371/journal.pone.0145780 (PMC4706341; doi:10.1371/journal.pone.0145780)
Supplement: S1 File — Bcl-xl (Figure A). Bax (Figure B). BAD (Figure C). Cytochrome C (Figure D). ERK 1/2 (Figure E). β-actin (Figure F). (PPTX) [file pone.0145780.s001.pptx]

## Slide 1
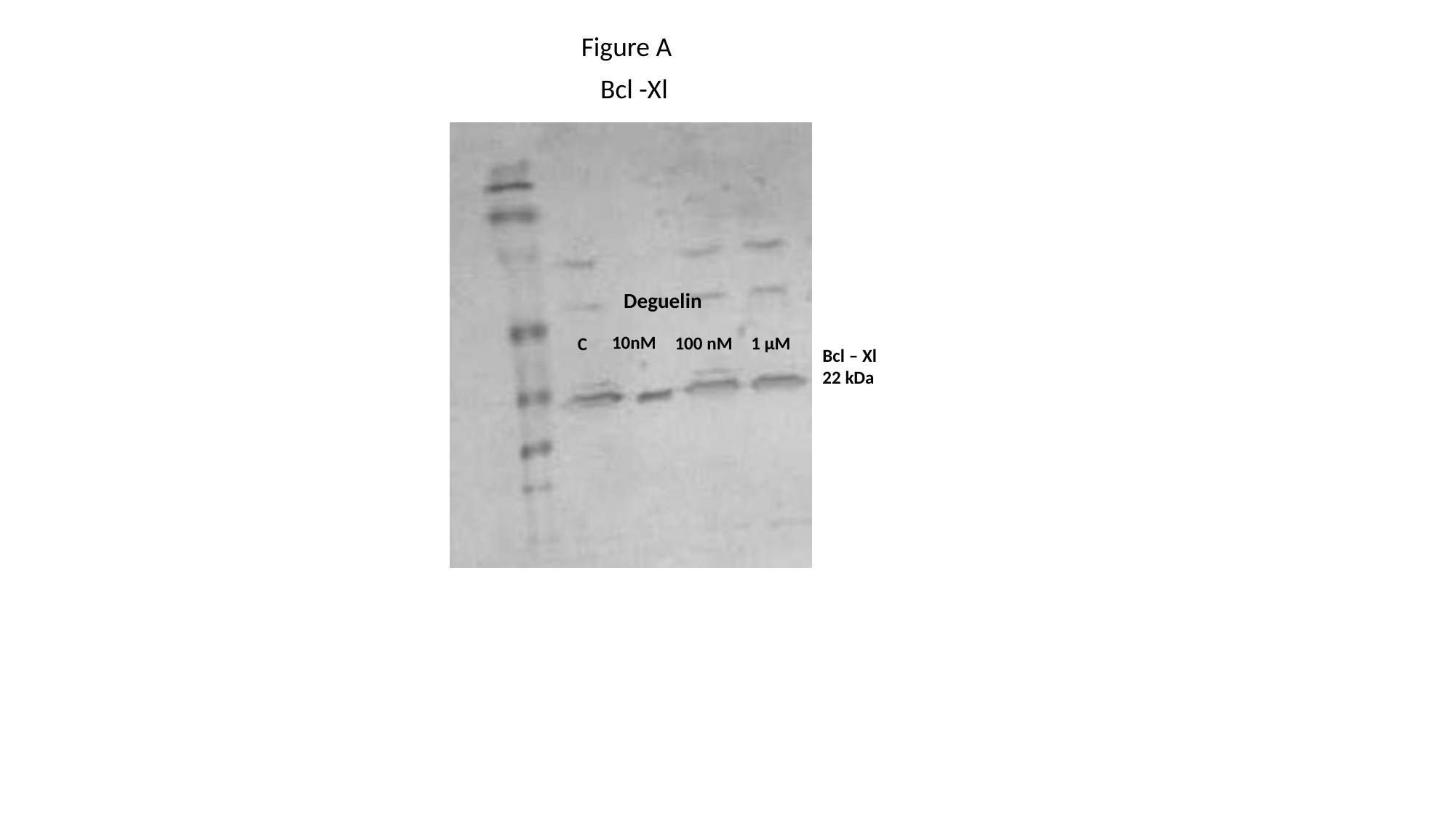

Figure A
Bcl -Xl
Deguelin
 10nM
 100 nM
 1 µM
 C
Bcl – Xl
22 kDa

## Slide 2
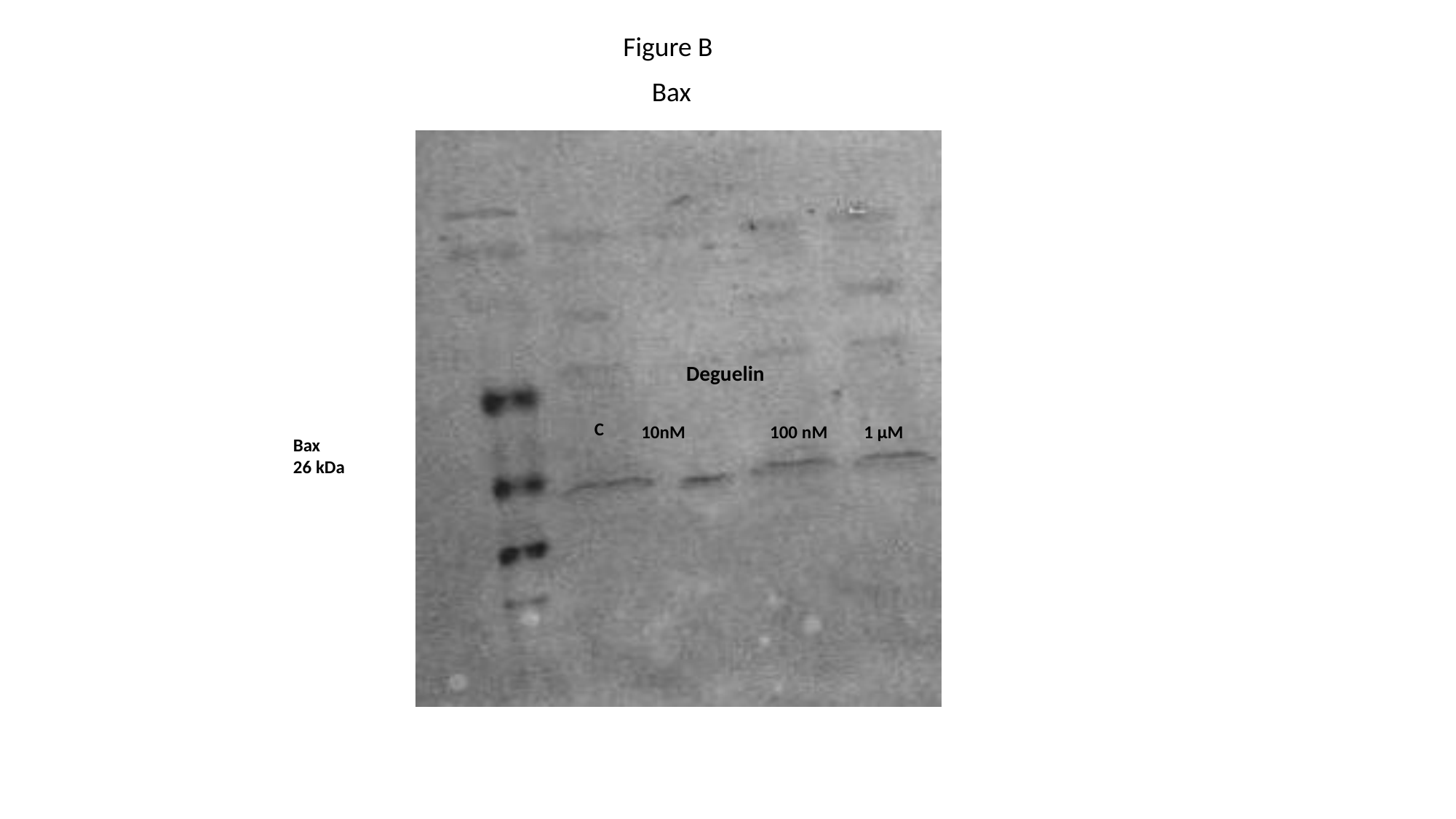

Figure B
Bax
Deguelin
C
10nM
100 nM
1 µM
Bax
26 kDa

## Slide 3
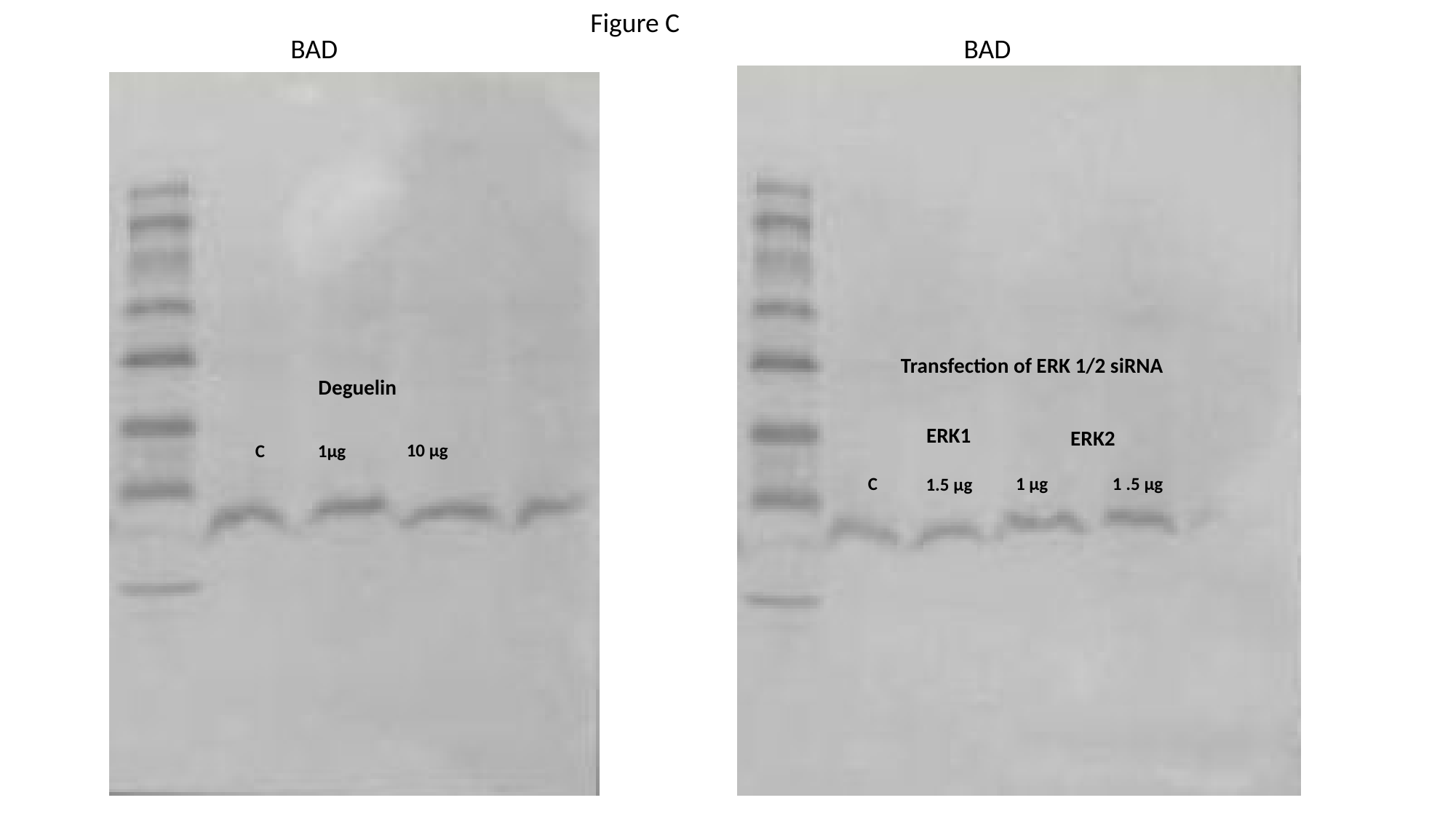

Figure C
BAD
BAD
ERK1
C
1 µg
 1 .5 µg
1.5 µg
ERK2
Deguelin
10 µg
C
1µg
Transfection of ERK 1/2 siRNA

## Slide 4
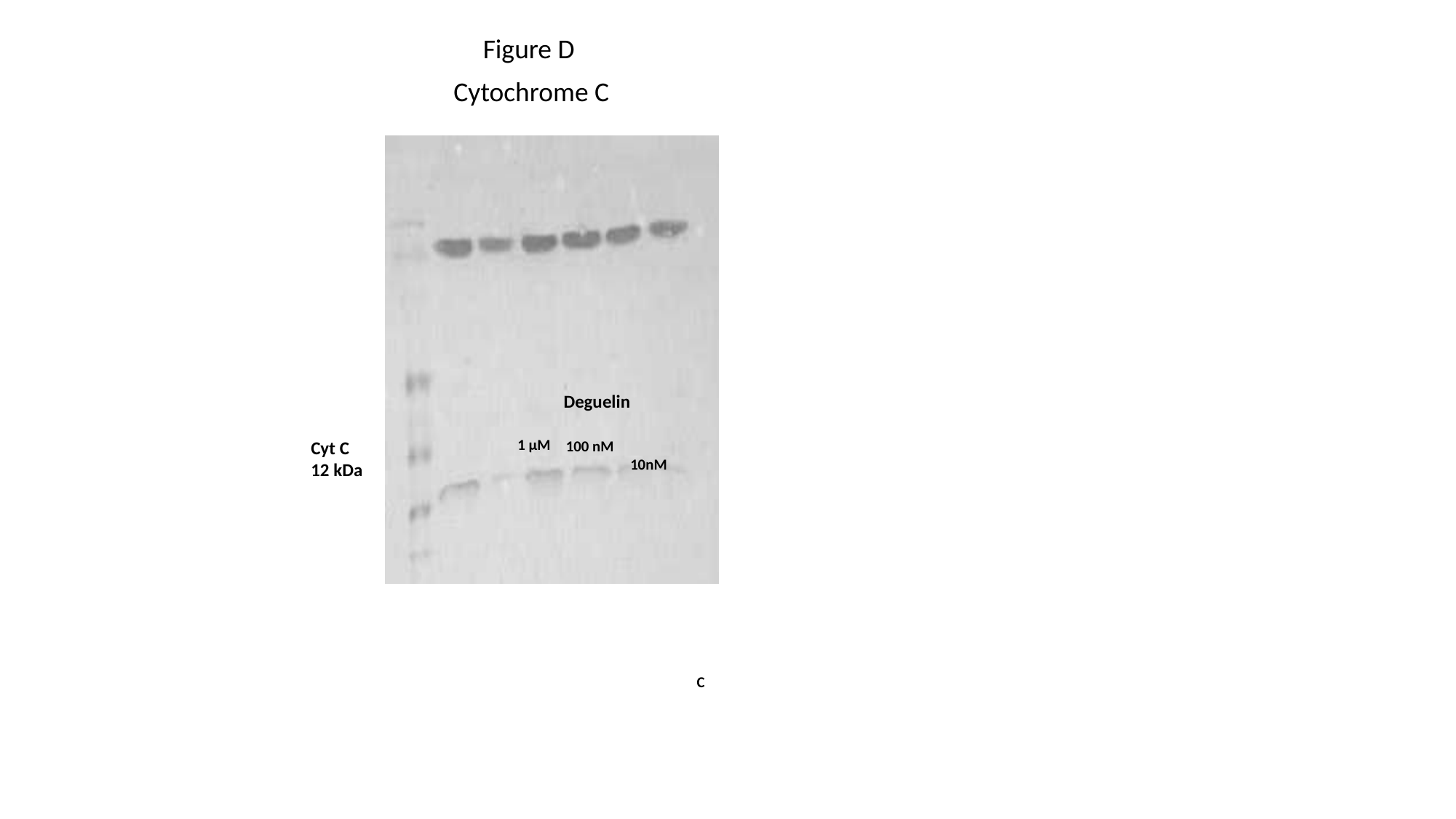

Figure D
Cytochrome C
Deguelin
 10nM
 C
 100 nM
 1 µM
Cyt C
12 kDa

## Slide 5
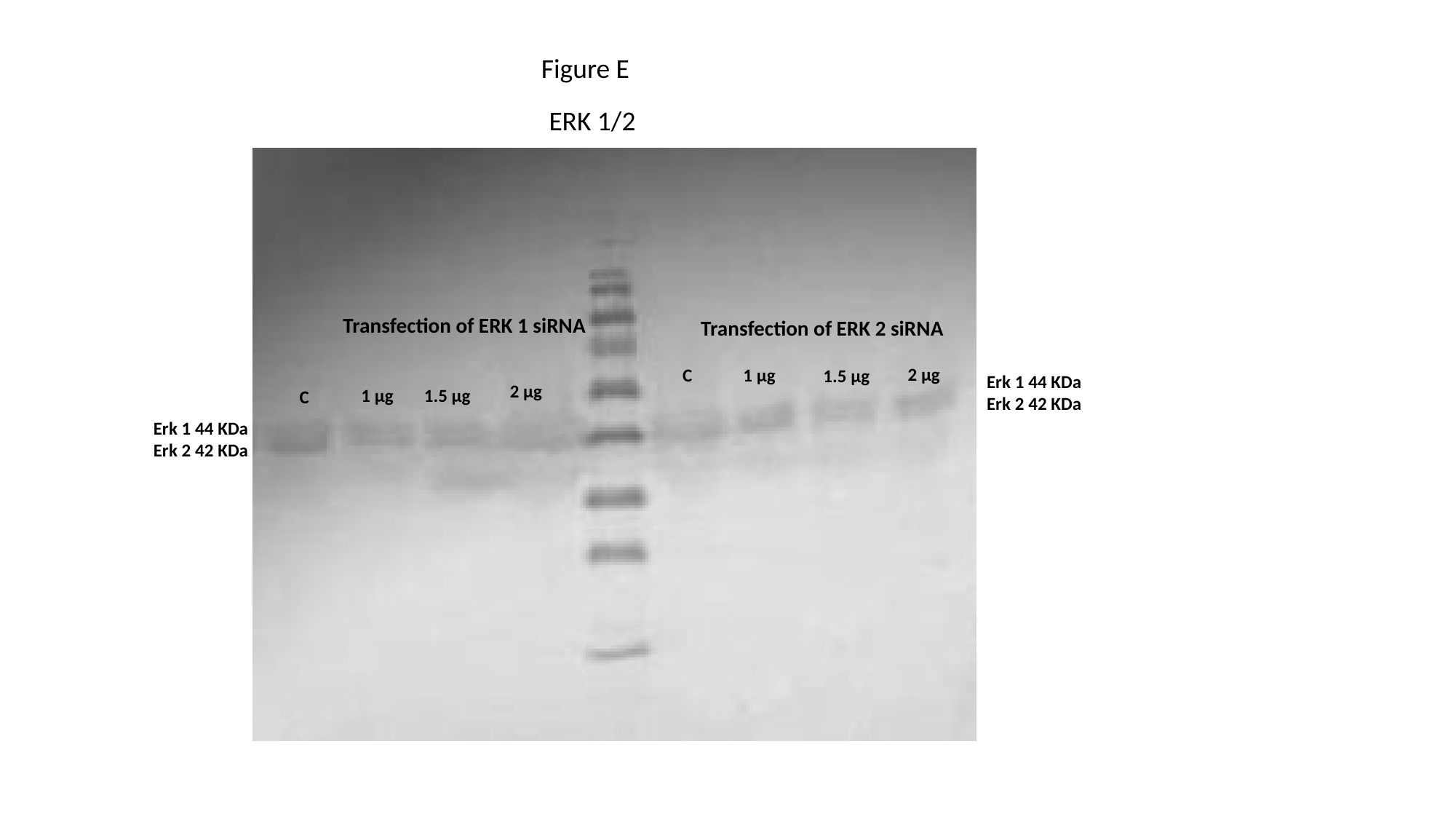

Figure E
ERK 1/2
Transfection of ERK 2 siRNA
2 µg
C
1 µg
1.5 µg
C
Erk 1 44 KDa
Erk 2 42 KDa
Transfection of ERK 1 siRNA
Erk 1 44 KDa
Erk 2 42 KDa
2 µg
1 µg
1.5 µg

## Slide 6
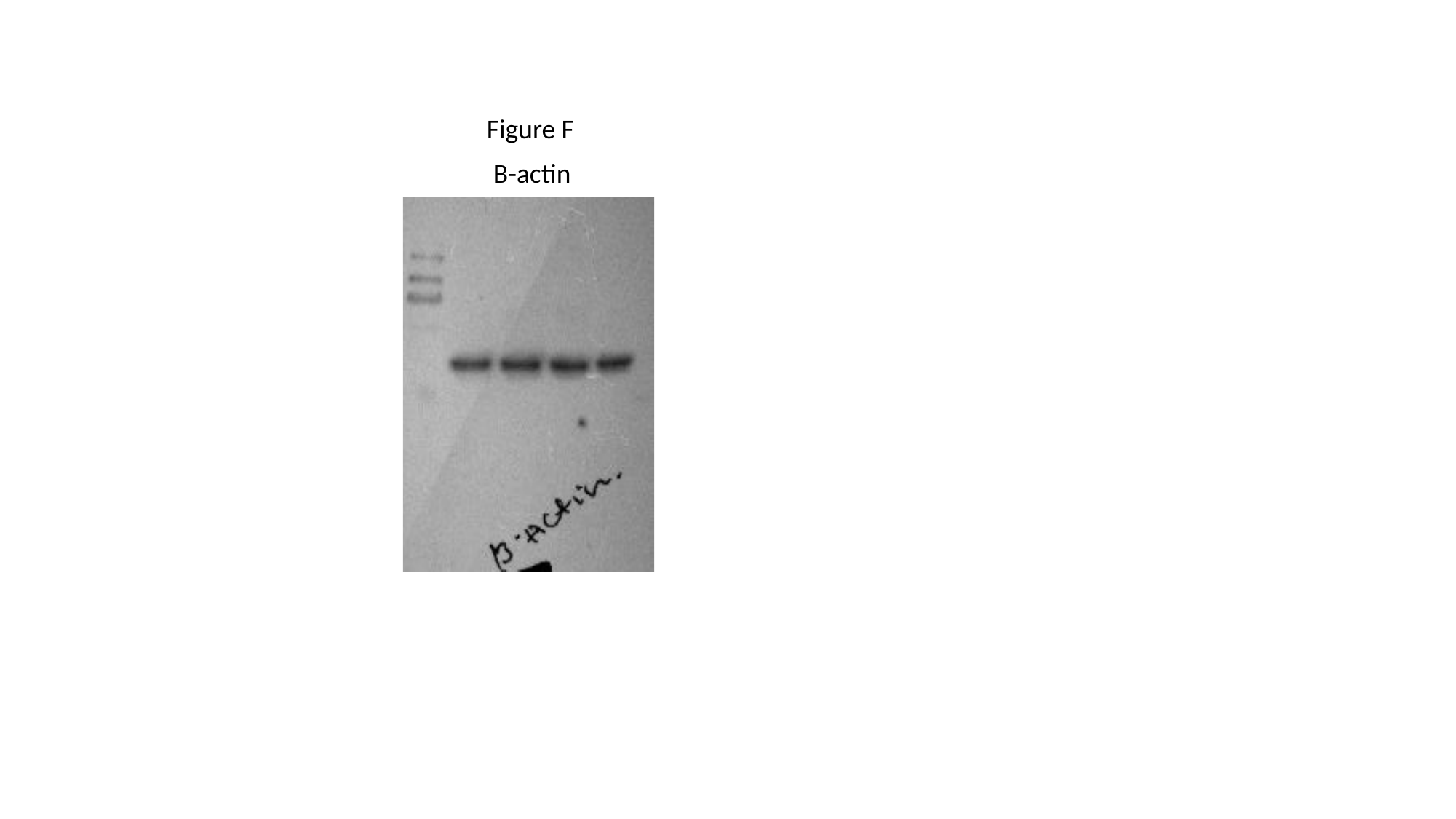

Figure F
Β-actin
